# Revisiting fall armyworm population movement in the United States and Canada

**DOI:** 10.3389/finsc.2023.1104793

**Published:** 2023-02-24

**Authors:** Ashley E. Tessnow, Rodney N. Nagoshi, Robert L. Meagher, Shelby J. Fleischer

**Affiliations:** ^1^ Department of Entomology, Texas A&M University, College Station, TX, United States; ^2^ U.S. Department of Agriculture- Agriculture Research Service- Center for Medical, Agricultural, and Veterinary Entomology (USDA-ARS CMAVE), Gainesville, FL, United States; ^3^ Retired, State College, PA, United States

**Keywords:** *Spodoptera frugiperda*, migration, genetic markers, population admixture, pest management

## Abstract

**Introduction:**

Biophysical approaches validated against haplotype and trap catch patterns have modeled the migratory trajectory of fall armyworms at a semi-continental scale, from their natal origins in Texas or Florida through much of the United States east of the Rocky Mountains. However, unexplained variation in the validation analysis was present, and misalignments between the simulated movement patterns of fall armyworm populations and the haplotype ratios at several locations, especially in the northeastern US and Canada, have been reported.

**Methods:**

Using an expanded dataset extending into Canada, we assess the consistency of haplotype patterns that relate overwintered origins of fall armyworm populations to hypothesized dispersal trajectories in North America and compare the geographic distribution of these patterns with previous model projections.

**Results and discussion:**

We confirm the general accuracy of previous modeling efforts, except for late in the season where our data suggests a higher proportion of Texas populations invading the northeast, extending into eastern Canada. We delineate geographic limits to the range of both overwintering populations and show that substantial intermixing of the Texas and Florida migrants routinely occurs north of South Carolina. We discuss annual variation to these migratory trajectories and test the hypothesis that the Appalachian Mountains influence geographic patterns of haplotypes. We discuss how these results may limit gene flow between the Texas and Florida natal populations and limit the hereditary consequences of interbreeding between these populations.

## Introduction

Migration allows organisms to exploit novel habitats that may not be suitable across all seasons, thus giving rise to seasonal variability in the spatial distribution of a species (1, 2). While this strategy is common across many taxa, the migratory patterns and behaviors of insects are only beginning to be understood (3), despite their importance for human interests, including agricultural pest management. Characterizing the migratory patterns of insect agricultural pests could allow for better identification and management of source populations, potentially reducing the number of migrants and protecting crops at more northern latitudes. This could also lead to better predictive models linking environmental factors in the overwintering habitats to a pest’s seasonal distribution. These pest distribution models or ‘forecasts’ have long been accepted as an important foundation for effective management (4).

The fall armyworm, *Spodoptera frugiperda* (J.E. Smith), is a caterpillar pest native to the western hemisphere, and invasive across Africa (5), Asia (6–8) and Australia (9). This polyphagous species, with 353 host plants recorded (10), is known to cause substantial damage to crops including corn, sorghum, and pasture grasses, with more occasional losses reported in millet, alfalfa, rice, vegetable crops, and cotton (11, 12). The fall armyworm species is comprised of two morphologically identical but genetically distinct strains, the C-strain and the R-strain, that co-occur throughout the Americas. These strains are host associated (13–15) or more recently allochronic (16–18), referring to the two primary behavioral differences that have been observed between strains. For the purposes of this study, we will focus on the C-strain which is a primary economic pest of corn and sorghum.

Fall armyworm overwintering survival is limited to areas that enable development of immature life stages year-round. In North America, fall armyworms are only able to survive the winter at two locations; southern Florida and the southern tip of Texas (11); in the absence of freeze events, the northern limit in Florida has been estimated to be between 28 and 29^°^N (19). These overwintering locations divide the fall armyworm species into two unique geographic populations, henceforth referred to as the Florida population (FL) and the Texas population (TX). Each year as temperatures begin to rise in the spring, fall armyworm moths move north from these overwintering sites in a stepwise generational expansion. The long-range movement north from these overwintering locations was first inferred from trapping data across the continent (11), and later corroborated by meteorological simulations (20, 21). These simulations suggested that the TX population moved north into the central US, west of the Appalachian Mountains, while the FL population moved north, primarily east of the Appalachians with a subset of migrants moving west into the Mississippi Valley (20). However, these methods were not able to reliably determine which population was the source of migrants in the far north, or the extent to which these two populations mixed along the migratory path.

Sequence data of the *Cytochrome Oxidase I* (COI) mitochondrial gene has identified unique haplotype variants that can be used to determine the overwintering origin (TX or FL) of C-strain individuals (22). To identify the geographic origin of the C-strain individuals, two SNP variants present at two loci in the COI gene give rise to four possible haplotypes that can be found in both overwintering populations, with two (h2 and h4) most frequently observed. Importantly, the ratio of h2 and h4 differs between the TX and FL overwintering populations and has remained constant over time (23), indicating limited gene flow. Thus, the haplotype ratio of a population sampled at any location in North America can be used as a snapshot to identify the overwintering origin of the population at that location.

Previous studies have used these haplotype ratios and timing of first sustained trap capture as validation methods for models of fall armyworm moth movement throughout the continental US (24, 25). These simulations integrated the temperature-dependent progression of corn planting and development, life table approaches to capture the biology and phenology of fall armyworm on this corn host, and propensity of migratory behavior of fall armyworm. The fraction of the population that exhibited ascent migratory behavior was influenced by the developmental stage of the host plant experienced during the insect’s larval stage. Night-time air flow trajectory models were used to transport migratory adults for up to three nights post eclosion, and oviposition of transported moths was determined by deposition onto corn. Validation efforts over four years showed significant relations of the timing of simulated moth arrival with trap capture data, and the ratio of simulated TX and FL haplotypes with observed haplotype ratio. However, as in all simulations, unexplained variation in the validation analysis was present, and misalignments between the simulated movement patterns of fall armyworm populations and the haplotype ratios at several locations, especially in the northeastern US and Canada, have been reported.

Here, we present haplotype ratio data spanning a wider geographic region (extending into Canada) and longer temporal range (11 years) than previously assessed to revisit the dispersal trajectories of this species. Using this summary data, we address three specific objectives regarding fall armyworm movement in North America. First, we identify consistencies and discrepancies between current migration models and the haplotype ratios from our expansive collection data. Next, we assess the effects of the Appalachian Mountain Range on maintaining the genetic isolation between these two overwintering populations. Finally, we synthesize genotyping data, trapping data, and previous published observations to update the predicted migratory trajectory of this species as they disperse from the two overwintering locations.

## Methods

### Sample collection

Moth samples were collected between 2004 and 2015 using bucket-style traps (Unitraps, manufactured by International Pheromone Systems, Neston, UK, distributed by Great Lakes IPM, Vestaburg, MI, USA) baited with commercial pheromone lures and an insecticide strip. The number of C-strain moths collected and genotyped in each county for each year is listed in **Supplementary Table 1**. All moths were frozen at -20°C or immediately preserved in 95% ethanol and then shipped to the USDA-ARS Insect Behavior and Biocontrol Research Unit in Gainesville, FL, USA. Upon arrival, samples were stored at -20°C. Prior to molecular analysis, all samples were visually inspected to confirm their identity as fall armyworms.

### DNA isolation

To isolate DNA, samples were homogenized in 1.5 ml of phosphate buffered saline (PBS, 20 mM sodium phosphate, 150 mM NaCl, pH 8.0) using a tissue homogenizer (PRO Scientific Inc., Oxford, CT) or hand-held Dounce homogenizer then pelleted by centrifugation at 6000g for 5 min. at room temperature. The pellet was resuspended in 800 µl Genomic Lysis buffer (Zymo Research, Orange, CA) and incubated at 55°C for 15 min, followed by centrifugation at 12,000 rpm for 5 min. The supernatant was then transferred to a Zymo-Spin III column (Zymo Research, Orange, CA) to isolate genomic DNA according to the manufacturer’s instructions. Genomic DNA was stored at -20°C until sequencing.

### COI strain and haplotype determination

PCR amplification of the COI gene was performed as described previously (26). In brief, PCR reactions were conducted using 0.5 units Taq DNA polymerase (New England Biolabs, Beverly, MA), 3 μL of the corresponding 10× reaction buffer, 0.5 μL 10 mM dNTP, 0.5 μL 20 μM primer mix, 1–2 μL DNA template (between 0.05 and 0.5μg), and enough nuclease free water to bring the total reaction volume to 30ul. The primer pair used for this amplification was *CO1-893F* (5’-CACGAGCATATTTTACATCWGCA-3’) and *CO1-1472R* (5’-GCTGGTGGTAAATTTTGATATC-3’) which produce a 603-bp fragment containing an EcoRV site unique to the R-strain and two polymorphic loci at sites 1164 and 1287 that give rise to the R-strain haplotype (T_1164_A_1287_) and four unique C-strain haplotypes: CS-h1 (A_1164_A_1287_), CS-h2 (A_1164_G_1287_), CS-h3 (G_1164_A_1287_), and CS-h4 (G_1164_G_1287_). Primers were synthesized by Integrated DNA Technologies (Coralville, IA). The thermocycling program includes and initial denaturation at 94°C for 1 min, followed by 30 cycles of 92°C (30 s), 56°C (45 s), 72°C (45 s), and a final DNA extension at 72°C for 3 min.

To confirm strain identity, 5units of the restriction enzyme EcoRV (New England Biolabs), 4 μL of the manufacturers 10× restriction enzyme buffer, were added to the initial 30ul PCR product. Nuclease free water was used to bring the final reaction volume to 40ul. This reaction was then incubated at 37°C for 1-3hrs. The final product was run on a 1.8% agarose gel containing GelRed (Biotium, Hayward, CA).

Since the R-strain contains a unique EcoRV cut site, there are two bands on the gel while the C-strain remains as a single band of 601bp. C-strain bands were extracted from the gel using the Zymoclean Gel DNA Recovery kit (Zymo Research) according to the manufacturers protocol. The isolated fragments were analyzed by Sanger sequencing using primers *CO1-893F* or *CO1-1472R* (University of Florida ICBR Center and Genewiz, South Plainfield, NJ). DNA sequences were uploaded to the DS Gene program (Accelrys, San Diego, CA, USA) or the Geneious 10.0.7 software (Biomatters, Auckland, New Zealand) to confirm strain identity and identify haplotypes.

### Haplotype analysis

Using the DNA sequence data of the C-strain individuals, *CO1* segment haplotypes were determined by assessing polymorphisms present at two distinct loci (1164 and 1287): h1 (A_1164_A_1287_), h2 (A_1164_G_1287_), h3 (G_1164_A_1287_), and h4 (G_1164_G_1287_). Two of these haplotypes are infrequent in North America (h1 and h3), whilst two are common (h2 and h4). The ratio of the two common haplotypes differs between the overwintering populations with h2 being predominant in TX, and h4 being predominant in FL (22). This ratio has remained constant overtime (23) and therefore has been used to determine the overwintering location of a sampled population. However, a simple ratio (h4/h2) is incalculable when there are no individuals in a collection that have the h2 haplotype. Therefore, we calculated a modified haplotype ratio using the formula (h4 –h2)/(h4 + h2), as has been previously described. Using this metric, the FL population has been defined as any collection with a ratio greater than or equal to 0.1 (with a maximum of 1.0 when h2 = 0), and the TX population was defined as any collection with a ratio less than or equal to -0.3 (with a minimum of -1.0 when h4 = 0) (26). All collections with values between -0.3 and 0.1 are then considered a “mixed” profile likely containing individuals from both the FL and TX populations. If a location had multiple collections that were inconsistent with either a TX or FL origin, these locations were also considered to be populated by a mix of individuals from both TX and FL. All locations for which we calculated the modified haplotype ratio and used the TX, FL, or mixed designations are presented in **Table 1**. The mean haplotype ratio at every location was then plotted, and standard error was presented for locations with multiple collection times. For locations that had multiple years of collection data, the mean haplotype ratio was presented for each year along with standard error bars when multiple collections were averaged within a year.

**Table 1:** Corresponding locations and collection years for all US and Canadian collections presented in **Figures 3**, **5**.

	Map #	Location	Collection Year
**Figure 3**	1	Saint-Arsene, QC	2015
	2	La Pocatiere, QC	2015
	3	L’Islet, QC	2015
	4	Saint-Gilles, QC	2015
	5	Louiseville, QC	2015
	6	Nicolet, QC	2015
	7	Saint-Ephrem-de-Beuce, QC	2015
	8	Compton, QC	2015
	9	Franklin, MA	2015
	10	Ridgetown, ON	2011 & 2013
	11	Erie, PA	2011-2015
	12	Suffolk, NY	2011-2015
	13	Centre, PA	2011 & 2013-2015
	14	Camden, NJ	2013
	15	Cape May, NJ	2011-2015
**Figure 5**	1	Eastern Maryland	2012
	2	Roanoke, VA	2011-2015
	3	Suffolk, VA	2011-2012
	4	Winslow, NC	2004
	5	Clayton, NC	2006
	6	Henderson, NC	2008
	7	Moore, NC	2004
	8	Brunswick, NC	2004
	9	Charleston, SC	2011-2015

### Haplotype data compilation, visualization and statistical analyses

Because low sample sizes can significantly skew haplotype ratios, we pooled collections that occurred in the same season within a single year, as in previous modeling efforts (24, 25). Moths that were collected between August and October of 2011 to 2015 were pooled as fall collections. If there were less than 10 individuals collected at a single location after pooling, we did not include that location in our analysis. A full factorial generalized linear model followed by a Tukey’s HSD posthoc test was used to assess the effects of year and mountain range side (east vs west of the Appalachian Mountains) on the *S. frugiperda* modified haplotype ratios at each collection location in the fall season (August to October). Only years 2011-2015 had sufficient collection data both east and west of the Appalachian Mountains to assess this hypothesis, therefore these were the only years included in the model.

Prior to running this model, data were checked for normality using the Shapiro-Wilk test, and variances were confirmed to be homogenous using the Brown-Forsythe test. Statistical analyses were run in JMP 16.1.0 (SAS Institute Inc., Cary, NC).

In Suffolk, VA strain data was reported each month (July to October) from 2012 to 2015. Therefore, Pearson’s correlation was run in R 4.0.5 to assess if there was a significant correlation between the proportion of each strain in the population and the modified haplotype ratio of the C-strain individuals.

Graphical representations of the monthly mean wind vectors for the summer months of June, July and August, using the 1991-2020 time period, were pulled from the International Research Institute for Climate and Society (iri.columbia.edu), using their interface at https://iridl.ldeo.columbia.edu/maproom/Global/Climatologies/Vector_Winds.html. We used the 925.0 mb pressure setting, which is similar to the isobaric vertical motion setting used in the HYSPLIT air flow trajectory method used in the biophysical simulations of fall armyworm migration (24, 25).

All maps presented in this manuscript were made in qGIS (27).

### Trap capture data analysis from across Pennsylvania

Trap capture data from across Pennsylvania for the years 2012-5 was downloaded from the EDDMaps data repository (28, https://www.eddmaps.org/). Three counties that bracket the Appalachians, Erie County in the northwest, and Lancaster and York Counties in the southeast, had consistent trap capture reporting for all years and thus were selected for further analyses. Cumulative trap capture (CTC) was calculated by continuously summing the total number of moths captured to date within each county. CTC was then plotted against day of year (DOY) in JMP (29). This was done both by combining data across all years and by splitting the data by year.

The number of reported moths captured across all three counties was 6,960 (Erie= 6,719, Lancaster= 50 and York=191). The proportion of total moths that were captured in each respective county by each DOY was calculated as p(CTC) = (CTC)/6960.

This value was then linearized using a logit function ln(p(CTC)/1-p(CTC)). After transformation, the logit(p(CTC)) was linearly related to DOY in all counties for a 92-day period (DOY 183-275) at the peak of the trapping season (Jul 1-Oct 1). A linear regression was then fit to assess the heterogeneity of slope and intercept across the three different counties. Significant differences in the slope parameter estimate between counties indicates significantly different trap capture rates.

## Results

### Annual variation in haplotype ratios

**Figure 1:**
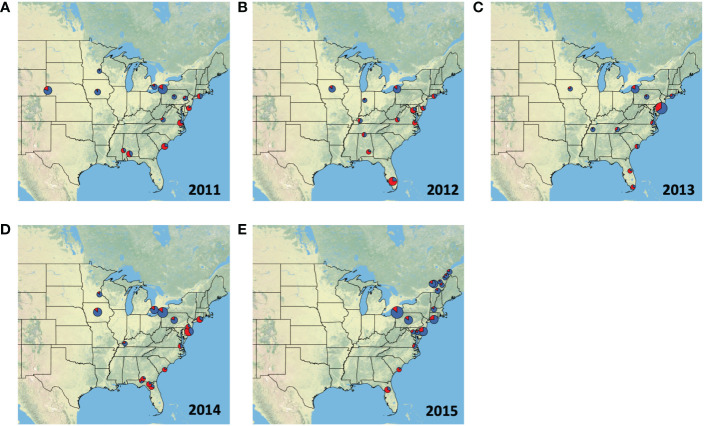
Proportion of individuals from each fall collection (August, September & October) with the h2 (red) or h4 (blue) COI haplotype in **(A)** 2011, **(B)** 2012, **(C)** 2013, **(D)** 2014, and **(E)** 2015. The size of each pie chart is scaled to represent the number of samples collected in each location. Collections information is also listed in **Table 1**. Texas populations are known to be dominated by h4 haplotypes, whereas the h2 haplotype is more abundant in the Florida populations.

**Figure 2:**
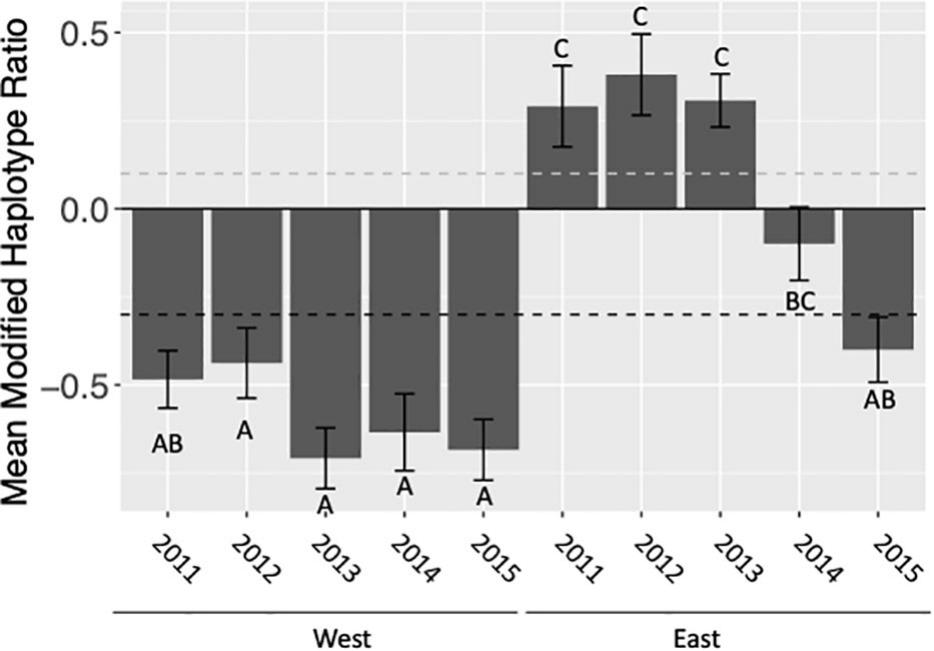
Mean modified haplotype ratio calculated as (h4-h2)/(h4+h2), pooled for all locations, of C-strain fall armyworm populations collected east and west of the Appalachian Mountain range in the fall (August-October). Ratios above the dashed grey line are representative of the FL population. Ratios below the dashed black line are representative of the TX population. Letters indicate statistically significant differences between mean haplotype ratios among years.

We compiled data on haplotypes from a total of 4,209 moths, distributed among 46 sites between 2004 and 2015 (**Supplementary Table 1**). As can be expected from a migratory species, we only had sufficient data across all locations in the fall season, therefore **Figures 1**, **2**, and **Table 2** only display data from the fall (August to October). Raw haplotype ratios from August to October in years ranging from 2011 to 2015 are plotted in **Figure 1**. This summary data indicates that populations in Florida and Georgia continue to be made up primarily of individuals with the h2 haplotype, while the population in the central US and Midwest are dominated by moths that carry the h4 haplotype. However, large annual variation exists in the proportion of h2 to h4 haplotypes present in collections that occur in the northeastern US. Collections in 2012 and 2013 were dominated by h2 haplotypes while collections in 2014 and 2015 overwhelmingly consisted of h4 haplotypes. Populations in eastern Canada, which we assessed in 2015, were also comprised overwhelmingly of the h4 haplotype. This data indicates that locations in New York, New Jersey, Maryland, Virginia, and North Carolina have the potential to be occupied by both the FL and TX populations, but the extent to which these populations invade is subject to annual fluctuations.

**Table 2:** Assessing the effects of year (2011-2015) and mountain range side (east vs west of the Appalachian Mountains) on the *S. frugiperda* modified haplotype ratios.

Source	DF	F Ratio	p-value
Year x MtnSide	4	3.84	0.0060**
Year	4	8.02	<0.0001***
MtnSide	1	108.34	<0.0001***

**0.01 > p <0.0001.

***p <0.0001. There was a significant interaction between the year and the side of mountain range where a collection occurred. All collections assessed in this comparison occurred in the fall (August-October) due to lack of sufficient data from any other season.

### Appalachian Mountains act as a barrier to gene flow

When assessing the effects of location with respect to the Appalachian Mountain range (east vs. west), year, and the year by location interaction on the haplotype ratio of collected populations our full factorial ANOVA model was significant (F_9,105 =_ 18.41, p<0.0001). Additionally, all effects − mountain range side, year, and their interaction − were significant (**Table 2**). This indicates that the Appalachian Mountains do limit geneflow between populations. However, the extent to which this geological barrier limits geneflow varies annually. This variation is always the result of an increase in h4 haplotypes in the east, which was most apparent in 2014 and 2015, rather than an increase in h2 haplotypes detected in the west (**Figure 2**).

### FAW population composition in the northeast and Canada

**Figure 3:**
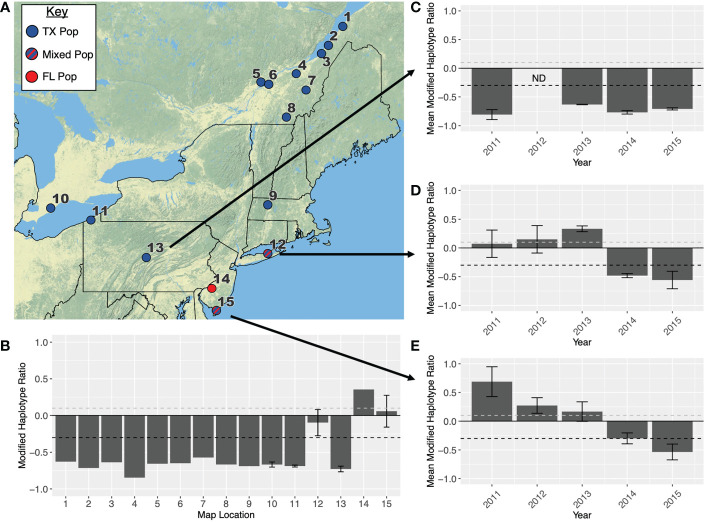
**(A)** Map showing the primary overwintering population that infests each location in the northeastern USA. All location names are listed in **Table 1**. Red indicates that a population originates in Florida, blue indicates a population originates in Texas, and blue-red stripes indicate a population is admixed between both overwintering sites. **(B)** Modified haplotype ratios (h4-h2)/(h4+h2) calculated across all fifteen locations. Additionally, modified haplotype ratios are presented for **(C)** Centre County, PA **(D)** Suffolk, NY and **(E)** Cape May, NY across the years 2011-2015. When locations had more than one collection (i.e., different months), ratios across locations were averaged and error bars indicate SEM. Every ratio above the dashed grey line on each graph is representative of the FL population, and every point below the dashed black line is representative of the TX population.

The northeastern US is largely dominated by individuals from the TX overwintering population (**Figures 3A, B**; **Table 1**), which arrive in this area from the west. This is especially true in Pennsylvania (**Figure 3C**), and regions north of New York and into Canada. There is evidence that Long Island, NY, Cape May, NJ, and Camden, NJ, which are along the eastern seaboard, receive some fall armyworm moths from the FL population (**Figures 3D, E**; **Table 1**), but when the TX population arrives from the west, they overwhelm the migrant population coming in from the south. Annual variation in the size of the population arriving from the west likely determines which overwintering population is dominant in these regions.

### Trap captures are much higher in northwest Pennsylvania compared to southeast Pennsylvania

**Figure 4:**
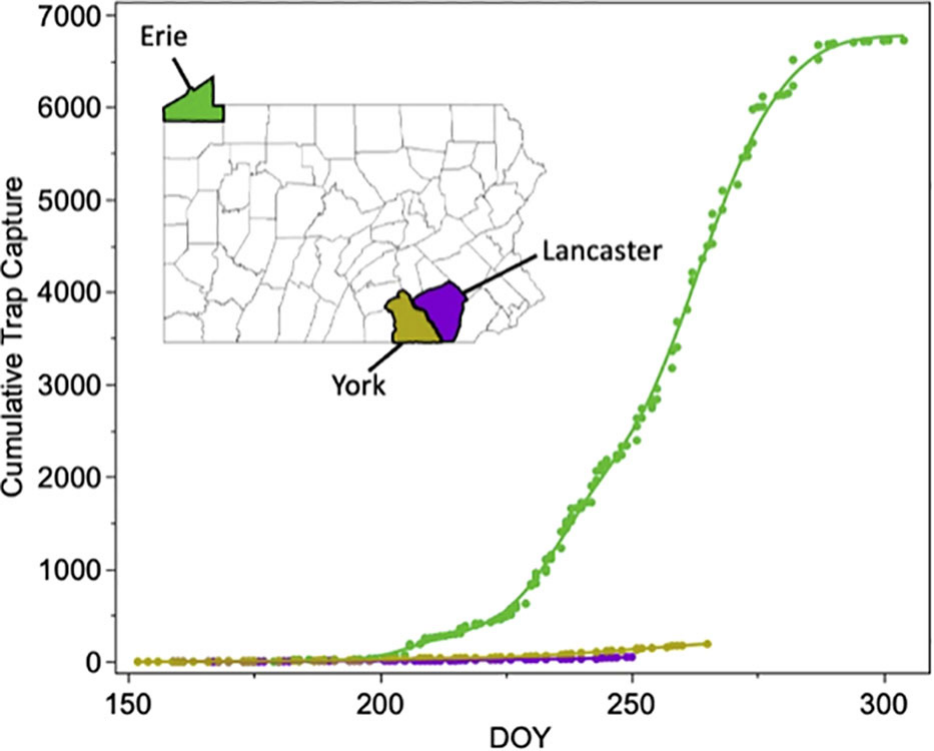
Cumulative trap capture plotted against day of year for three counties in Pennsylvania that bracket the Appalachia mountains. Capture data is summed across 2012-2015. Capture data supports a large source of fall armyworms arriving from the west in the northwest corner of the state.

Analysis of the Pennsylvania trap capture data from Erie County, York County, and Lancaster County in 2012-15 indicates that fall armyworm populations are much larger in northwestern Pennsylvania than in southeast Pennsylvania at all timepoints throughout the year (**Figure 4**). Our linear regression model indicated that DOY (F_1,1 =_ 4183.6, p<0.001), County (F_2,2 =_ 4851.0, p<0.001) and DOY x County (F_2,2 =_ 575.8, p<0.001) were all significant predictors of cumulative trap capture. When conducting pairwise comparisons between each of the three counties, both the slope and intercept were significantly different for each county comparison (**Supplementary Figure 1C**). This data confirms that northwestern Pennsylvania is receiving fall armyworm moths primarily from the west rather than from the southeast, consistent with previous trap surveys (30).

### FAW population composition in the southeast

**Figure 5:**
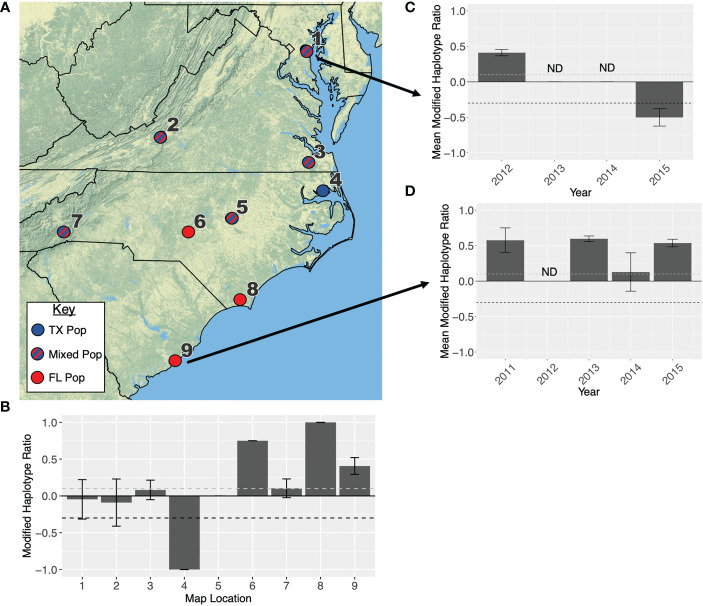
**(A)** Map showing the primary overwintering population that infests each location in the southeastern USA. Location names are listed in **Table 2**. Red indicates that a population originates in Florida, blue indicates a population originates in Texas, and blue-red stripes indicate a population is admixed between both overwintering sites. In 2004, only 8 moths were collected each from Moore and Winslow counties in North Carolina, therefore these locations were not included in any statistical analyses. **(B)** Modified haplotype ratios are presented for all nine collection locations. When locations had more than one collection (i.e., collections in different months or years), the ratios were averaged between collections and error bars indicate standard error. If no error bars are presented, this bar represents a single collection. **(C)** Modified haplotype ratios from eastern Maryland across years. **(D)** Modified haplotype ratios across years in Charleston, SC. Every ratio above the dashed grey line on each graph is representative of the FL population, and ratios below the dashed black line are representative of the TX population.

While the northeast is largely dominated by the TX population, the southeastern states are more variable. An influx of migrant individuals from both the TX and FL populations seems to occur regularly in Maryland, Virginia, and North Carolina (**Figure 5**; **Table 1**). Our collection data indicates North Carolina is the southernmost limit to the TX population east of the Appalachians, with some annual variation to the intermixing in this state (**Figure 5C**). The TX population was never reported in South Carolina, at least not in large enough numbers to move the haplotype ratio (**Figure 5D**).

**Figure 6:**
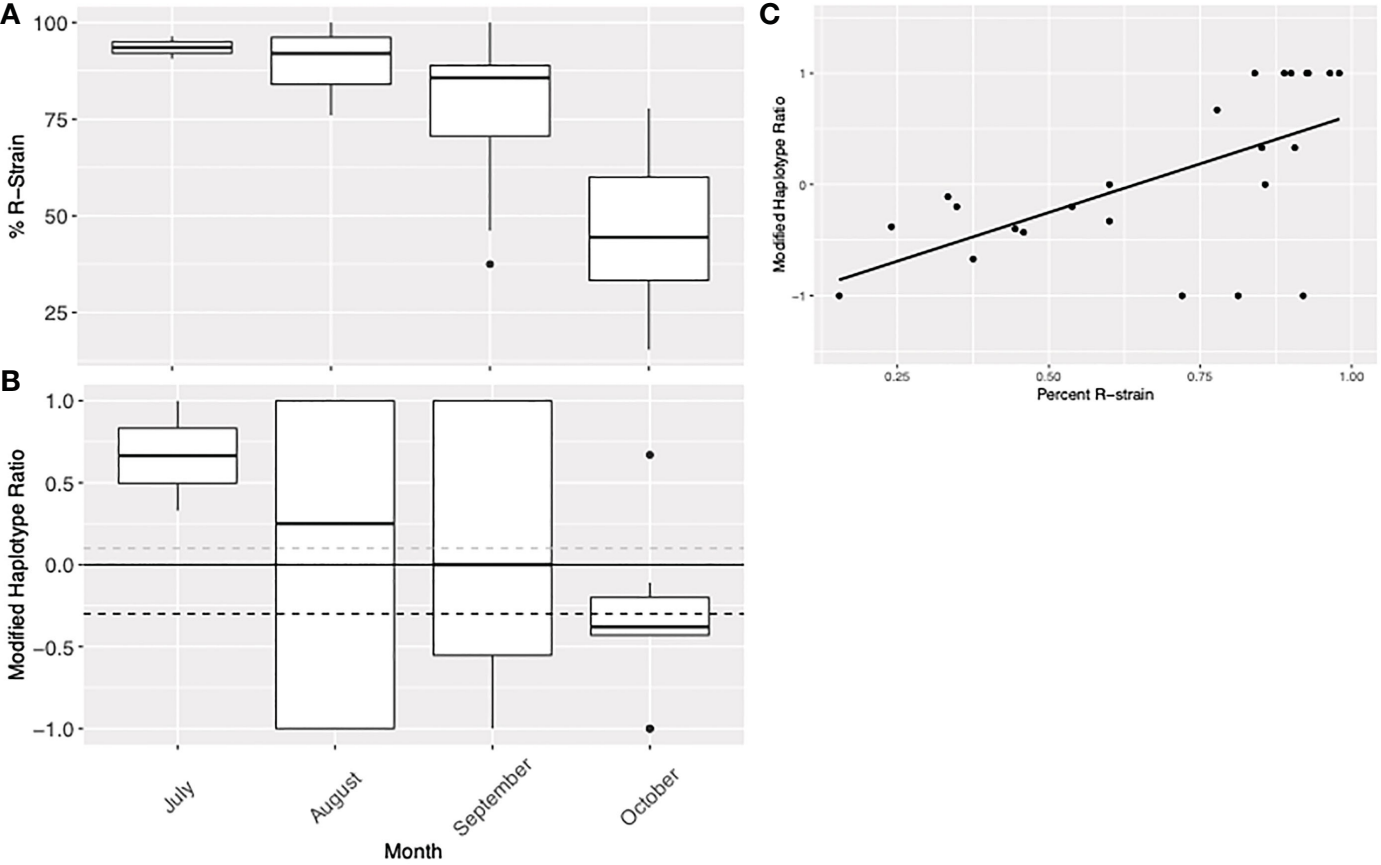
Visual representation of Virginia collection data from 2012 to 2015 demonstrating the **(A)** proportion of individuals collected which were identified as R-strain and **(B)** the modified haplotype ratio of C-strain individuals. This data indicates there may be a temporal pattern where C-strain individuals from the TX population arrive later in the season, shifting both the strain demographics and the haplotype ratio. **(C)** Linear correlation between the percent of R-strain individuals in the population and the modified haplotype ratio of the C-strain individuals. Because of the nature of this data, there are fewer C-strain samples assessed as the percent R-strain increases, so variables are not independent.

Data from Suffolk, VA in 2012 to 2015 suggested that the R-strain dominated collections early in the season (July & August), but the C-strain became more predominant in September and October. This corresponded with a decrease in the modified h2/h4 ratio of the C-strain population. This indicates a temporal pattern where C-strain individuals from the TX population arrive later in the season, shifting both the strain demographics and the haplotype ratio (**Figures 6A, B**). This is evidenced by the simultaneous decrease in both the proportion of R-strain individuals in the population, and the modified haplotype ratio of the C-strain population collected later in the season. The data was not normally distributed (Shapiro Wilk, p= 0.00836), so a Pearson’s correlation was used to show that these variables were linearly correlated (**Figure 6C**, t = -3.6055, df = 22, p-value = 0.001571). This pattern was only observed in Suffolk, VA, as no other collection had enough data about both strains and haplotype ratio over multiple consecutive months to assess this trend.

**Figure 7:**
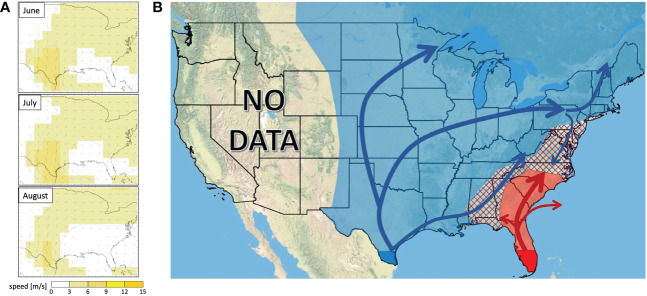
**(A)** Wind vector maps showing average wind directions at 925.0 mb across the continental US in June, July, and August in years 1991-2020. **(B)** Hypothesized dispersal trajectory for fall armyworms originating at the TX population (blue arrows) and the FL population (red arrows). Overwintering sites are highlighted in dark blue (TX) and red (FL). Annual invasion ranges are highlighted in light blue for the TX population and light red for the FL population with intermixing zones represented by hashmarks.

Mean wind vectors for the summer months (**Figure 7A**) show a northerly flow from Texas, shifting to a consistent west-to-east trajectory across the mid to northern portion of North America east of the Rocky Mountains. Wind trajectories in the southeast tend to be anticyclonic and more variable.

## Discussion

Using an expanded dataset of fall armyworm moths trapped across eleven years and over a larger geographic range that previously assessed, our data was largely consistent with previous models of fall armyworm movement across North America (24, 25), demonstrating the consistency of the North American FAW migratory pathway over time. Specifically, our haplotype data agrees with the hypothesis that the population that overwinters in south Texas (TX) spreads north and east to dominate the central US west of the Appalachian Mountain Range. Additionally, our data confirm that the population that overwinters in south Florida (FL), disperses north, east of the Appalachian Mountains as has been previously described (14, 24, 25).

One of the primary differences that we observed between our haplotype survey and previous migration modeling occurs in the northeastern US where simulations projected migrants from FL would predominate over those from TX in late summer (25). In contrast, our haplotype data indicates this region is primarily comprised of individuals from the TX population with little evidence of a detectable contribution from FL migrants in central Pennsylvania, Massachusetts, or Canada. Areas with haplotype ratios indicative of a significant FL component were limited to the coastal areas of New Jersey and New York, with the northern limit occurring in Long Island, NY.

There are a few possible explanations for this observation. First, the original migration models used HYSPLIT for air trajectories and relied on isobaric vertical motion, which should deflect migration over high-elevation mountain ranges. Although preliminary modeling using isentropic vertical motion options failed to advance the simulated migrations through the growing season, future efforts with variable vertical motions may refine projected haplotype distributions. Also, the density of the source populations from TX and FL may influence model results throughout the season. Despite limitations in the northeast, the fall armyworm migration simulations were accurate across locations in the south and central US. These models are still useful for predicting general pest population trends such as the timing of movement from the southern overwintering sites, the relative intensity of pest populations, and the shifts in pest population dynamics due to changing climactic conditions.

The Appalachian Mountains have previously been implicated in separating the fall armyworm population from Florida from that in Texas (23), and our current genetic data confirms that this range may act as a semi-permeable barrier, especially at northern latitudes. Since, the elevation of the Appalachians tends to decrease as you move from south to north, a proportion of the TX population seem to cross this range at more northern latitudes. Still, our trapping data suggests that this fraction is low, with nearly 270x more moths being collected in a single county in northwestern Pennsylvania than in two southeastern counties combine. Lower numbers of FL fall armyworms may also be moving into this region from the south than were predicted by previous modeling efforts, with a proportion of this population possibly being swept into the Atlantic Ocean by westerly winds (**Figure 7A**). Combined, these two scenarios would generate a small TX dominated fall armyworm population in the northeast, as was observed. Further work to evaluate the contribution of wind patterns explaining FAW migration patterns could include higher resolution of haplotyped samples coupled to more detailed analysis of wind patterns during those specific space and time periods, such as circular statistics which would provide both vector means and variability, or numerical trajectory modeling approaches (31).

Additional factors other than elevation, such as river topography and lack of suitable host plants, may also be limiting the TX population from crossing the Appalachian range during their multigenerational migration. In the southeast, the Flint River has been shown to act as a barrier between the FL and TX populations (32), and therefore it is possible that similar geographic features may contribute to maintaining the isolation between the TX and FL population in the northeast. However, given that large rivers, such as the Mississippi River, do not seem to impede FAW movement in the central US, it remains unclear why and how these smaller geographic features influence fall armyworm migration.

Additionally, in the northeast, much of this area is forested, and newly arrived immigrants may not establish in sufficient numbers due to lack of food resources. In more southern areas, relatively little corn acreage is planted at high elevations in western North Carolina, Virginia, and West Virginia (24). The C-strain TX populations may simply be expanding their range in conjuncture with the availability of food resources. While the original models did attempt to incorporate corn acreage into the distribution predictions (24) and corn is a favored host plant, the C-strain is known to be polyphagous and may exploit secondary plant hosts in these locations.

Emerging evidence also indicates that previous migratory models may oversimplify moth directional movement. While past modeling efforts have assumed that moth movement has been driven primarily or exclusively by wind direction (20, 21, 24, 25), recent studies in other nocturnally migrating moths including the sphingid, *Acherontia atropos* (L.), and the noctuid, *Mythimna unipuncta* (Haworth), indicate that nocturnally migrating moths may have more control over their dispersal trajectory than previously thought (33–35). Additionally, in the more well studied lepidopteran, the monarch butterfly (*Danaus plexippus* (L.)), orientation behavior has been shown to vary seasonally, influenced by environmental conditions (36, 37), facilitating northward migration early in the season, followed by southward migration later in the season. These active seasonal orientation mechanisms could lead to discrepancies between the aerobiology modeling and the genetic data and should be further assessed to better determine the migration trajectories of the fall armyworm.

Our current dataset also indicates that the zone of admixture between the FL and TX population is much larger than previously predicted, with the TX population expanding as far south as North Carolina in some years. We detect intermixing near coastal areas in New York and New Jersey, and into Maryland, Virginia, and North Carolina. There was never enough TX population samples collected in South Carolina to alter the haplotype ratio, possibly indicating a southernmost limit to the TX population. We propose that after crossing the Appalachian range in Pennsylvania in July, some of the TX population then move south, east of the Appalachians. This is supported by data from Suffolk, VA, where R-strain and C-strain individuals from FL are primarily detected in July and August, but by September and October, traps consist almost entirely of C-strain individuals from TX. The southernmost extent to this intermixing zone varies annually but does not appear to move beyond SC even during the years with high levels of southward introgression by TX individuals. This suggests that few (if any) descendants of the northeastern FAW populations return to FL overwintering sites, indicating that the mixing of the TX and FL migrants in the northeast are of little hereditary consequence as these do not contribute to subsequent generations.

Annual variation in the intermixing zone was also evident in the northeast. Here, in 2011-2013, the TX population arrived in Pennsylvania but was not detected south of this state. However, in 2014-2015, the TX population once again arrived in high numbers in Pennsylvania but showed a substantial spread further south. These observations could result from either increased migration from TX (relative to FL) in 2014-5 or conversely reduced migration from FL during this time period. There are indications that both could be the case. In 2011-3, the Lower Rio Grande Valley in Texas experienced drought conditions possibly reducing the overall size of the overwintering fall armyworm population (National Integrated Drought Information System) (38). However, in 2014-15 precipitation was above average in this region, potentially leading to a larger initial population moving north and eventually invading the northeast. This is similar to observations in monarch butterflies where precipitation in the Texas spring breeding grounds is the greatest predictor of midwestern population size later in the season (39). With respect to the FL migration, corn acreage in South Carolina in 2014-5 was reduced by 10%-18% compared to 2011-13, which could have led to lower migratory numbers of C-strain fall armyworm from FL to the northeastern states (**Supplementary Table 2**).

Using both our current dataset and previous work delimitating the ranges of the FL and TX population in the southeast (32), we updated the hypothesized fall armyworm migration map (**Figure 7B**). Wind patterns largely aligned with our observed distribution of TX and FL haplotypes, with summer wind climatology (e.g., monthly vector-means at 925-mb or an approximate altitude of 762m) showing a north and east flow in the central continental US, turning to a west-to-east vector across the northern part of this area (**Figure 6A**). Most nocturnal moth migration occurs between 200-800m, (40, 41), so this is a good indication of the wind currents that moths experience during migratory flight. Active navigation by fall armyworm moths should be further explored as a possible explanation for discrepancies between the wind trajectories and the hypothesized moth movement patterns.

Although these haplotype ratios can be a good indication of population level movement from the overwintering sites, there are some limitations to this methodology. First, these haplotype ratios cannot be used to pinpoint the origin of any individual moth. Since the h2 and h4 haplotypes are present in both the FL and TX populations, an individual with either haplotype could have originated in either overwintering location. As a result, a large sample is required to determine the likely overwintering location of a population. Additionally, these haplotype ratios may not be good indicators of overall population level admixture. If a few migrants from one population (TX) mix with a large flight from the other population (FL), the additional haplotypes may not alter the haplotype ratio above the set thresholds, so this admixture would not be detected. Additionally, these haplotype ratios are limited to C-strain fall armyworms, and thus do not provide information on R-strain individuals. Therefore, these markers should be considered low resolution, providing a limited snapshot into the larger continental scale movement patterns of this species.

In the future higher resolution markers for both strains, such as single nucleotide polymorphisms (SNPs), should be considered to better track population movement from the overwintering sites. Previous SNP data found that the FL and TX population exhibit a low-level reduction in gene flow between populations (42), but no population specific SNP markers have been identified to date. Further exploring genetic differentiation between these two overwintering populations for both C- and R-strains fall armyworms may improve the migratory map, and better identify regions of admixture between these two populations as they invade more northern latitudes.

Population dynamics of noctuids in areas north of their overwintering locations are influenced by source populations (43). Understanding these linkages is key to IPM strategies targeting source areas and projecting the effects of climate change. Developing tools to forecast fall armyworm movement and population dynamics is key to refining IPM strategies, especially in the eastern US that can be inhabited by either overwintering population, and the Midwest where the large corn acreage can be put at risk. Understanding the movement patterns of this species across North America can provide early warnings to better manage impending pest attacks (44). This could also lead to improved models that predict where outbreaks in the migratory regions may occur, based on environmental conditions at the overwintering locations. Finally, tracking the continental scale seasonal movement of fall armyworm moths will provide a baseline for future investigations into how climate change may alter this species distribution (45), and how this may impact crop protection plans.

## Data availability statement

The original contributions presented in the study are included in the article/**Supplementary Material**. Further inquiries can be directed to the corresponding author.

## Author contributions

AT contributed to the data analysis and writing of this manuscript. RM organized sample collections and edited the manuscript. RN genotyped all samples and edited the manuscript. SF contributed to sample collections, data analysis, and writing of the manuscript. RM, RN, and SF conceived of the project and obtained funding. All authors contributed to the article and approved the submitted version.
