# Supplementary material for: Revisiting fall armyworm population movement in the United States and Canada
Source: Front Insect Sci. 2023 Feb 24;3:1104793. doi: 10.3389/finsc.2023.1104793 (PMC10926481; doi:10.3389/finsc.2023.1104793)
Supplement: Supplementary file 2 [file Table_1.docx]

**Supplementary Table 1.** Number of samples collected and genotyped per year from each of the collection locations. The individual who collected from each location is listed.

|  |  | **Sample Size by Year** | | | | | | | |  |
| --- | --- | --- | --- | --- | --- | --- | --- | --- | --- | --- |
| **County** | **State** | **2004** | **2006** | **2008** | **2011** | **2012** | **2013** | **2014** | **2015** | **Collector** |
| Butler | AL |  |  |  | 36 |  |  |  |  | R. Meagher |
| Conecuh | AL |  |  |  | 118 |  |  |  |  | R. Meagher |
| Limestone | AL |  |  |  |  | 13 |  |  |  | R. Meagher |
| Pike | AL |  |  |  |  | 13 |  |  |  | R. Meagher |
| Alachua | FL |  |  |  |  |  | 192 | 14 |  | R. Meagher |
| Belle Glade | FL |  |  |  |  |  | 7 |  |  | R. Meagher |
| Hendry | FL |  |  |  |  | 122 |  |  |  | R. Meagher |
| Hillsborough | FL |  |  |  |  | 13 |  |  |  | R. Meagher |
| Marion | FL |  |  |  |  |  |  |  | 64 | R. Meagher |
| Martin | FL |  |  |  |  | 7 |  |  |  | R. Meagher |
| Miami-Dade | FL |  |  |  |  | 15 |  | 207 |  | R. Meagher |
| Orange | FL |  |  |  |  | 56 |  | 28 |  | R. Meagher |
| Palm Beach | FL |  |  |  |  | 19 |  | 66 |  | R. Meagher |
| Suwannee | FL |  |  |  |  |  | 62 | 5 |  | R. Meagher |
| Decatur | GA |  |  |  |  |  | 17 |  |  | R. Meagher |
| Mitchell | GA |  |  |  |  |  | 31 |  |  | R. Meagher |
| Seminole | GA |  |  |  |  | 10 |  |  |  | R. Meagher |
| Spalding | GA |  |  |  |  |  |  | 13 |  | R. Meagher |
| Tift | GA |  |  |  |  |  | 19 |  |  | R. Meagher |
| Polk | IA |  |  |  | 66 |  |  |  |  | T. Sappington |
| Story | IA |  |  |  |  | 79 | 126 | 57 |  | T. Sappington |
| Tippecanoe | IN |  |  |  |  | 19 |  |  |  | J. Obermeyer/R. Foster |
| Calwell | KY |  |  |  |  | 19 | 11 |  |  | P. Lucas/D. Johnson |
| Henderson | NC |  |  | 155 |  |  |  |  |  | J. Walgenbach |
| Clayton | NC |  | 20 |  |  |  |  |  |  | C. Sorenson |
| Moore | NC | 8 |  |  |  |  |  |  |  | D. Reisig |
| Brunswick | NC | 11 |  |  |  |  |  |  |  | D. Reisig |
| Winslow | NC | 8 |  |  |  |  |  |  |  | D. Reisig |
| Franklin | MA |  |  |  |  |  |  |  | 50 | R. Hazzard |
| Beltsville | MD |  |  |  |  |  |  |  | 33 | G. Dively |
| Prince Georges | MD |  |  |  |  | 57 |  |  |  | G. Dively |
| Queen Anne | MD |  |  |  |  |  |  |  | 17 | G. Dively |
| Rosemont | MN |  |  |  | 16 |  | 52 |  |  | E. Burkness |
| Scottsbluff | NE |  |  |  | 117 |  |  |  |  | J. Bradshaw |
| Camden | NJ |  |  |  |  |  | 32 |  |  | J. Ingerson-Mahar |
| Cape May | NJ |  |  |  | 46 | 33 | 135 | 187 | 176 | K. Holmstrom |
| Suffolk | NY |  |  |  | 38 | 44 | 77 | 62 | 186 | D. Gilrein |
| Ridgetown | ON |  |  |  | 52 |  | 104 |  |  | T. Baute |
| Centre | PA |  |  |  | 36 |  | 77 | 55 | 187 | S. Fleischer |
| Erie | PA |  |  |  | 150 | 101 | 178 | 165 | 283 | A. Muza |
| Compton | QC |  |  |  |  |  |  |  | 64 | B. Duval/ J.P. Légaré |
| L'Islet | QC |  |  |  |  |  |  |  | 12 | B. Duval/ J.P. Légaré |
| La Pocatiere | QC |  |  |  |  |  |  |  | 16 | B. Duval/ J.P. Légaré |
| Louiseville | QC |  |  |  |  |  |  |  | 76 | B. Duval/ J.P. Légaré |
| Nicolet | QC |  |  |  |  |  |  |  | 109 | B. Duval/ J.P. Légaré |
| Saint-Arsene | QC |  |  |  |  |  |  |  | 47 | B. Duval/ J.P. Légaré |
| Saint-Ephrem-de-Beuce | QC |  |  |  |  |  |  |  | 14 | B. Duval/ J.P. Légaré |
| Saint-Gilles | QC |  |  |  |  |  |  |  | 13 | B. Duval/ J.P. Légaré |
| Charleston | SC |  |  |  | 63 |  | 25 | 41 | 63 | A. Simmons |
| Blount | TN |  |  |  |  |  |  | 22 |  | W. Klingeman |
| Madison | TN |  |  |  |  |  |  | 18 |  | F. Hale |
| Blacksburg | VA |  |  |  |  |  | 6 |  |  | T. Kuhar |
| Montgomery | VA |  |  |  |  |  |  | 18 |  | T. Kuhar |
| Roanoke | VA |  |  |  | 17 | 15 |  |  |  | T. Kuhar |
| Suffolk | VA |  |  |  | 60 | 11 | 33 | 72 | 27 | T. Kuhar |
