# Supplementary material for: Revisiting fall armyworm population movement in the United States and Canada
Source: Front Insect Sci. 2023 Feb 24;3:1104793. doi: 10.3389/finsc.2023.1104793 (PMC10926481; doi:10.3389/finsc.2023.1104793)
Supplement: Supplementary file 3 [file Table_2.docx]

**Supplementary Table 2.** Acres of corn planted in six states across the five-year period when haplotypes were assessed for this study. There was a notable decrease in corn acreage in the Carolinas in 2014 and 2015 (red). This data was retrieved from: [https://quickstats.nass.usda.gov/](https://nam10.safelinks.protection.outlook.com/?url=https%3A%2F%2Fquickstats.nass.usda.gov%2F&data=05%7C01%7CAshley.Tessnow%40ag.tamu.edu%7Ccf0c77b5d9ac43c6e40708dab7886430%7C9fd7580a64724d9ca142d131d3a7a116%7C0%7C0%7C638024093672929662%7CUnknown%7CTWFpbGZsb3d8eyJWIjoiMC4wLjAwMDAiLCJQIjoiV2luMzIiLCJBTiI6Ik1haWwiLCJXVCI6Mn0%3D%7C3000%7C%7C%7C&sdata=kJNuavwUrEG9oCmAp73Us%2B8cKZMSL0NHjEeoe28eD5c%3D&reserved=0).

|  | **2011** | **2012** | **2013** | **2014** | **2015** |
| --- | --- | --- | --- | --- | --- |
| **FL** | 70,000 | 75,000 | 115,000 | 75,000 | 80,000 |
| **GA** | 345,000 | 345,000 | 510,000 | 350,000 | 330,000 |
| **NC** | 870,000 | 870,000 | 930,000 | 840,000 | 790,000 |
| **SC** | 360,000 | 330,000 | 350,000 | 295,000 | 295,000 |
| **PA** | 1,420,000 | 1,460,000 | 1,480,000 | 1,460,000 | 1,340,000 |
| **TX** | 2,050,000 | 1,850,000 | 2,350,000 | 2,250,000 | 2,300,000 |
