# Supplementary material for: Revisiting fall armyworm population movement in the United States and Canada
Source: Front Insect Sci. 2023 Feb 24;3:1104793. doi: 10.3389/finsc.2023.1104793 (PMC10926481; doi:10.3389/finsc.2023.1104793)
Supplement: Supplementary file 4 [file Image_1.pdf]

(a)

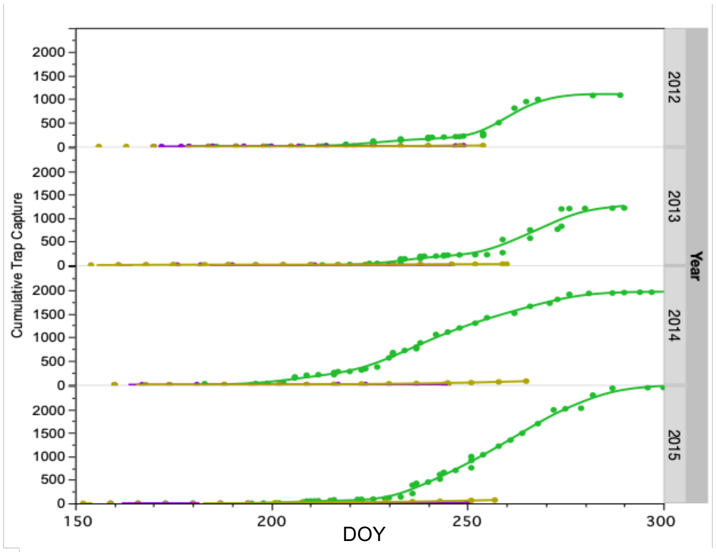

(b)

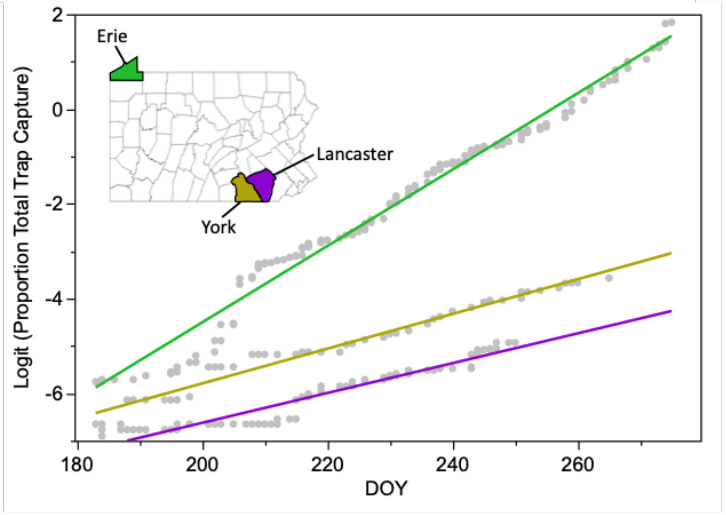

(c)

	Intercept		Slope	
**County Comparison**	**t-ratio**	**p-value**	**t-ratio**	**p-value**
Erie vs. Lancaster	90.4	<0.001**	27.46	<0.001**
Erie vs. York	61.51	<0.001**	26.12	<0.001**
Lancaster vs. York	-20.39	<0.001**	-2.44	0.015*

Supplementary Figure 1. (a) Cumulative fall armyworm trap capture plotted against day of year (DOY), split across years. (b) Linearization of cumulative trap capture plotted against day of year for three counties in PA; Erie County (green), Lancaster County (purple), and York County (gold). (c) Pairwise statistical differences in linear regression slope and intercept parameter estimates for Erie, Lancaster, and York counties. Significant differences indicate trap capture rate significantly differed between the two compared counties.
